# Regulation of Dietary Habits: The effect of losing weight on quality of life

**DOI:** 10.12669/pjms.345.15667

**Published:** 2018

**Authors:** Ibrahim Bashan, Mustafa Bakman, Yucel Uysal, Ertan Mert

**Affiliations:** 1 Ibrahim Bashan, MD. Department of Medical Education, Faculty of Medicine, Mersin University, Mersin, Turkey; 2 Mustafa Bakman, MD. Toroslar Ay Isigi Primary Care Center, Mersin, Turkey.; 3 Yucel Uysal, MD. Department of Family Medicine, Faculty of Medicine, Mersin University, Mersin, Turkey; 4 Ertan Mert, MD. Department of Family Medicine, Faculty of Medicine, Mersin University, Mersin, Turkey

**Keywords:** Dietary habit, Quality of Life, Questionnaires

## Abstract

**Objective:**

This study aimed to investigate the effects of regulation of nutritional habits on quality of life by applying Short Form (SF-36) Quality of Life Scale.

**Methods:**

SF-36 was administered through face-to-face interviews to a total of 129 individuals (112 female, 17 male) aged 18-65 years. Anthropometric measurements, body mass index, and waist circumference measurement were undertaken simultaneously. The changes in weight, body mass index, waist circumference measurement, and SF-36 scores were compared at baseline and three months after modification of dietary habits.

**Results:**

At three months after the regulation of dietary habits, a statistically significant decrease was found in weight, body mass index, and waist circumference measurements (p<0.05). Overall improvement was observed in all the quality of life parameters assessed with SF-36, and among them, the sub-scales of general health, bodily pain and vitality were statistically significant (p<0.05). The improvement in these sub-scale scores was similar to the literature.

**Conclusion:**

The individuals who lost weight through adopting healthy dietary habits had increased SF-36 scores, indicating the positive effect of regulating diet on their quality of life.

## INTRODUCTION

Nutrition-related health problems, such as obesity adversely affects the quality of life in both direct and indirect ways.^1^,^2^ In order to preserve, maintain and improve the quality of life, the nutritional intake should be sufficient and balanced.^3^ Much like the diseases related to malnutrition, obesity and related problems that are caused by unbalanced and overfeeding affect the quality of life negatively.^4^-^6^

Quality of life is an improving general parameter of the comfort level of patients.^7^ It is a multi-factor concept formed by the individual perception of physical, psychological and social functioning, and the measurement of quality of life is becoming increasingly important in many areas of health research.^8^,^9^

The Short Form Quality of Life Scale (SF-36), which was adopted into Turkish by Kocyigit H et al. and proved to be reliable and valid^10^, is one of the most frequently used quality of life scales in the medical area and consists of 36 items structured under eight sub-scales. The distribution of items according to the sub-scales is as follows: physical functioning (10 items), social functioning (2 items), role limitations due to physical functioning (4 items), role mental (emotional problems) (3 items), mental health (5 items), vitality (4 items), bodily pain (2 items), and general health (6 items).^11^

In this study, we aimed to investigate the effect of dietary habit regulation on quality of life in overweight and obese individuals who were recommended a healthy diet program. For this purpose, we compared the SF-36 scores at baseline and three months after the regulation of dietary habits.

## METHODS

A total of 450 cases were included in this study, of which only 129 (112 female, 17 male) completed the SF-36 scale. SF-36 was introduced to the study population aged 18 to 65 years who applied to the Family Medicine Polyclinic of Mersin University Medical Faculty for regulation of their dietary habits and were recommended a healthy nutritional program by a single executive. The data was collected using the face-to-face interview technique at first visit and three months after the regulation of nutritional habits. Furthermore, anthropometric measurements (height and weight) were simultaneously undertaken, and body mass index (BMI), waist circumference measurement (WCM) change ratios and scale scores were calculated. Pregnant individuals, those that could not verbally communicate or did not have the mental capacity to understand or respond to the questionnaire, and those that did not agree to participate in the survey were not included in the study. This study was approved by the Medical Research Ethics Committee (approval date: 28/05/2015 and number: 2015/166).

### Statistical Analysis

The SPSS (version 15.0) software package was used for statistical analyses. The averages and correlation levels between the scores in relation to the different parameters were calculated by the paired-samples t-test, and the significance of the differences between the averages was checked by the Pitman-Morgan test. p<0.05 was considered to be statistically significant.

## RESULTS

The mean age of 112 women (86.8%) and 17 men (13.2%) of the 129 cases were found to be 39.4 ± 11.4 years (range 19-62), and the most frequent age range was 41-50 years (36.4%) (Table-I).Three months after the regulation of the dietary habits, there was a decrease of 4.7 kg in weight and 1.7 kg/m^2^ in BMI on average, and WCM was reduced by 4.9 cm in in women and 5.4 cm in men. The decrease in weight, BMI and WCM was statistically significant compared to the baseline values (p <0.05) (Table-II).

**Table-I:** Distribution of participants according to gender and age groups.

Age Groups (%)	18-30 years (29.4)		31-40 years (16.3)		41-50 years (36.4)		51-65 years (17.9)		Total (100)	
*(Mean ± SD)*	*n*	%*	*n*	%*	*n*	%*	*n*	%*	*n*	%*
Female (39.2±11.6)	35	92.1	16	76.2	42	89.4	19	82.6	112	86.8
Male (40.6±9.6)	3	7.9	5	23.8	5	10.6	4	17.4	17	13.2
Total (39.4±11.4)	38	100	21	100	47	100	23	100	129	100

**Table-II:** Average weight, BMI and WCM of the participants.

	n	At baseline	After three months of diet	p
Weight	129	85.3 ± 14.3 (61.0-131.0)	80.6 ± 13.5 (58.0-119.0)	p<0.05
BMI	129	31.4 ± 4.5 (23.7-44.5)	29.7 ± 4.4 (22.0-40.9)	p<0.05
WCM (Female)	112	101.5 ± 9.7 (82.0-126.0)	96.6 ± 9.6 (79.0-120.0)	p<0.05
WCM (Male)	17	110.8 ± 10.0 (99.0-128.0)	105.4 ± 9.4 (92-120)	p<0.05

When the quality of life scores were evaluated at baseline and three months after the dietary habit regulation, improvements were observed in all sub-scales of SF-36 at the end of the study. It was found that the sub-scale scores of general health, bodily pain and vitality were statistically significant compared to the baseline (p <0.05) (Table-III).

**Table-III:** Comparison of the quality of life scores at baseline and after three months of diet.

Sub-scales	n	Baseline	After three months	p
General health	129	16.7 ± 3.8 (5-25)	17.3 ± 3.6 (8-25)	p<0.05
Physical functioning	129	26.0 ± 3.1 (16-30)	26.6 ± 3.1 (17-30)	p>0.05
Role physical	129	6.6 ± 1.5 (4-8)	7.0 ± 1.3 (4-8)	p>0.05
Role mental	129	4.6 ± 1.2 (3-6)	5.0 ± 1.1 (3-6)	p>0.05
Social functioning	129	7.7 ± 1.7 (3-10)	8.0 ± 1.7 (2-10)	p>0.05
Bodily pain	129	7.7 ± 2.8 (2-10)	8.5 ± 2.0 (2-11)	p<0.05
Mental health	129	21.5 ± 3.8 (12-30)	21.9 ± 3.9 (11-30)	p>0.05
Vitality	129	14.2 ± 3.7 (4-22)	15.6 ± 3.5 (6-24)	p<0.05

## DISCUSSION

In a study investigating the effect of reduced weight on anthropometric measurements, biochemical data, and quality of life over six weeks with 22 obese female participants, Guclu LP^12^ reported a statistically significant decrease in weight, BMI and WCM, similar to the results of our study. Concerning the sub-scale scores of SF-36, Guclu LP et al.^12^ found a statistically significant increase in physical functioning, role physical, bodily pain, vitality, and general health, and significant decrease in the role mental scores at the end of the study. While there was a statistically insignificant decrease in mental health, no change was detected in social functioning scores. In the current study, the differences in the sub-scale scores of general health, bodily pain and vitality sub-scales statistically significant (p <0.05).While there was a significant decrease in the role mental sub-scale scores in Guclu LP’s study^12^, we found an insignificant increase in these scores. This may be due to the differences in the characteristics of the sample, which only consisted of obese female individuals in the study by Guclu et al.

In a 13-week study with 38 participants, Fontaine KR et al.^13^ evaluated the impact of weight loss on quality of life and found statistically significant improvements in general health, physical functioning, role physical, vitality, and mental health sub-scales at the end of this period.

In another study, overweight and obese patients were included in a clinical weight loss program targeting lifestyle changes (behavioral counseling, diet and exercise) and followed up for six, 12 and 24 months. Statistically significant improvements were observed in the physical functioning, general health, vitality and mental health sub-scales at the end of six and 12 months, and in physical functioning, vitality and mental health sub-scales at the end of 24 months.^14^

In this study, the improvement in the general health and vitality sub-scales was similar to the other studies. We found that three out of the eight sub-scales of SF-36 (general health, bodily pain, and vitality) improved significantly compared to the others. The reason for the fewer number of sub-scales producing significant results may be due to the lower number of participants and the shorter length of study.

Regulation of dietary habits, one of the lifestyle changes, increases the quality of life. It should be aimed to improve the health of overweight and obese individuals and help them gain healthy life styles to increase their quality of life. In addition to healthy eating, healthy lifestyle can be supported by undertaking regular physical activity and avoiding harmful habits, such smoking and alcohol consumption.

### Limitations of the study

Firstly, 129 of the 450 initial cases were able to complete the questionnaire both before and after regulation of dietary habits. Secondly, the majority of the participants were women, and finally, the duration of the study was relatively short (three months) compared to other studies in the literature.

### Author`s Contribution

**IB** conceived, designed, did statistical analysis & manuscript writing.

**MB** did data collection.

**YU** did statistical analysis & editing of manuscript.

**EM** did review and final approval of manuscript.
